# Characterization of Pressure Transients Generated by Nanosecond Electrical Pulse (nsEP) Exposure

**DOI:** 10.1038/srep15063

**Published:** 2015-10-09

**Authors:** Caleb C. Roth, Ronald A. Barnes Jr., Bennett L. Ibey, Hope T. Beier, L. Christopher Mimun, Saher M. Maswadi, Mehdi Shadaram, Randolph D. Glickman

**Affiliations:** 1 School of Medicine, Dept. of Radiological Sciences, University of Texas Health Science Center San Antonio, 7703 Floyd Curl Drive, San Antonio, Texas, USA 78229; 2 Dept. of Electrical Engineering, University of Texas San Antonio, 1 UTSA Circle, San Antonio, Texas, USA 78249; 3 Radio Frequency Bioeffects Branch, Bioeffects Division, Human Effectiveness Directorate, 711th Human Performance Wing, Air Force Research Laboratory, 4141 Petroleum Road, JBSA Fort Sam Houston, Texas, USA 78234; 4 Optical Radiation Bioeffects Branch, Bioeffects Division, Human Effectiveness Directorate, 711th Human Performance Wing, Air Force Research Laboratory, 4141 Petroleum Road, JBSA Fort Sam Houston, Texas, USA 78234; 5 Dept. of Physics, University of Texas San Antonio, 1 UTSA Circle, San Antonio, Texas, USA 78249; 6 School of Medicine, Dept. of Ophthalmology, University of Texas Health Science Center San Antonio, 7703 Floyd Curl Drive, San Antonio, Texas, USA 78229

## Abstract

The mechanism(s) responsible for the breakdown (nanoporation) of cell plasma membranes after nanosecond pulse (nsEP) exposure remains poorly understood. Current theories focus exclusively on the electrical field, citing electrostriction, water dipole alignment and/or electrodeformation as the primary mechanisms for pore formation. However, the delivery of a high-voltage nsEP to cells by tungsten electrodes creates a multitude of biophysical phenomena, including electrohydraulic cavitation, electrochemical interactions, thermoelastic expansion, and others. To date, very limited research has investigated non-electric phenomena occurring during nsEP exposures and their potential effect on cell nanoporation. Of primary interest is the production of acoustic shock waves during nsEP exposure, as it is known that acoustic shock waves can cause membrane poration (sonoporation). Based on these observations, our group characterized the acoustic pressure transients generated by nsEP and determined if such transients played any role in nanoporation. In this paper, we show that nsEP exposures, equivalent to those used in cellular studies, are capable of generating high-frequency (2.5 MHz), high-intensity (>13 kPa) pressure transients. Using confocal microscopy to measure cell uptake of YO-PRO®-1 (indicator of nanoporation of the plasma membrane) and changing the electrode geometry, we determined that acoustic waves alone are not responsible for poration of the membrane.

Nanoporation, a type of electroporation that generates very small (<2 nm) holes in plasma membranes, is hypothesized to result from exposures of sub-microsecond electric pulses in the megavolt/meter range^1^,^2^. The biophysical interactions that occur with an nsEP exposure are complex; therefore, determination of the mechanism of nanoporation is quite difficult. These biophysical interactions include, but are not limited to, electrothermal, electrochemical, electrohydraulic, electromechanical and electromagnetic phenomena. The conventional theory is that nanoporation occurs due to either electrostriction or electrodeformation of the plasma membrane. Electrostriction (altering the shape) of the plasma membrane is caused by the buildup of charge on the membrane leading to “pinching” of the phospholipids and thus pore formation^3^. Electrodeformation is an electrical-field-driven internal mechanical stress that causes the entire cell to deform, leading to a higher probability of pore formation^4^. Another competing theory of poration, championed by Vernier, has suggested poration occurs “due to field-induced reorganization of water dipoles at the water-lipid or water-vacuum interfaces”, presumably this reorganization of water molecules creates more energetically favorable situation for pore formation^5^. These theories of poration, although plausible, are not all-inclusive and do not account for other non-electrical factors, such as external mechanical stress caused by interactions with pressure transients.

Pressure transients have been shown to create pores in plasma membranes by imparting a mechanical stress^6^,^7^,^8^,^9^,^10^,^11^,^12^,^13^,^14^,^15^,^16^. Sonoporation uses ultrasonic waves (essentially pressure transients in the MHz range) to create holes in the biomembranes of cells and vesicles for the purposes of either delivering or releasing compounds, biomolecules, drugs, etc^8^,^10^,^13^. These ultrasonic shock waves can cause cavitation microbubbles, leading to poration by one of the following mechanisms: acoustic micro-streaming, bubble oscillations, or inertial cavitation shock waves^13^. Inertial cavitation shock waves, if of sufficient amplitude, impart mechanical stress on the plasma membranes of nearby cells leading to poration.

We hypothesize that pressure transients created by nsEP exposure^17^ are directly linked to the phenomena of nanoporation. We used the probe beam deflection technique (PBDT), an all-optical, non-contact method for detecting pressure transients generated in gaseous and liquid environments to characterize the pressure transients generated by typical nsEP expsoures^18^,^19^,^20^,^21^,^22^. With PBDT, the propagation of a pressure transient causes a change in the refractive index of the medium through which a probe beam travels, resulting in a deflection of that beam. This deflection is detected by a modified quadrature diode and quantified as the time derivative of a pressure transient. This approach is used in place of submerging a hydrophone in the conductive media, which is traditionally used to detect pressure transients but is not practical given the high-voltages consistent with nsEP. Further studies have also shown PBDT to be considerably more sensitive than most hydrophones, which are limited to their narrow cone of acceptance^23^.

We characterized the pressure transients based on frequency, amplitude, shape, and speed. We performed a Fast Fourier Transform (FFT) on pressure transient signals collected to determine the frequency of the pressure transients. We then used an ultrasonic transducer and a calibrated hydrophone to calculate the amount of pressure generated by nsEP exposure. In an effort to identify the source of the pressure transients, we used infrared thermography, Schlieren imaging, and pump-probe laser imaging to capture evidence of physical events occurring at or near the surface interface of the electrodes. Finally, we used confocal microscopy and the fluorescent dye YO-PRO(R)-1, to determine the effect of the pressure transients on nanoporation. The findings in this paper provide new insights as to the nature of the physical mechanisms that occur rapidly after the application of nsEP at the surface of the electrodes and how these events could potentially contribute to the breakdown of plasma membranes.

## Results

### Detection of Near-field Waves Produced by nsEP Using PBDT

**Figure 1:**
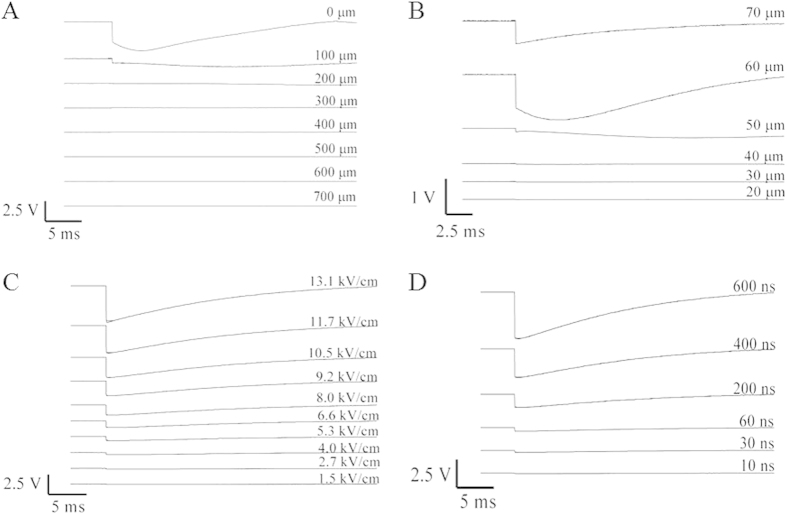
Near-field deflections detected by PBDT. (**A**) A collection of waveforms, each corresponding to PBDT deflection, captured as the electrodes were scanned in 100 μm increments up to 700 μm in the X+ plane. Maximum deflection of 2.0 V peak to peak was detected between 0 and 100 μm away from the probe beam. (**B**) These waveforms correspond to deflections captured in the Y+ plane in 10 μm increments up to 70 μm. The greatest deflection occurred at 60 μm from the probe beam. (**C**) These traces, collected in the X+ plane and 50 μm from the probe beam, show the linear response of input nsEP voltage (electric field) to PBDT deflection. The largest deflections were recorded with the highest electric field. No deflections where detected below 2.7 kV/cm. (**D**) These waveforms were collected from the same position as in 1C, and they show the relationship between pulse width on the resultant near-field deflection. The longer pulse widths give the largest deflections. At 30 ns, a small deflection can be detected.

When the electrodes were placed in very close proximity (<1 mm) to the probe beam, termed the near-field, substantial deflections of the probe beam were detected upon nsEP exposure. The nsEP exposure was administered with a pulse width of 600 ns and an applied voltage of 1000 V to generate an electrical field of approximately 13.1 kV/cm at 50 μm (typical cell exposure distance) from the electrodes. The electrical field strength was calculated in a FEM model/simulation for nsEP using the applied voltage measured on an oscilloscope as described in the methods section. The largest near-field deflections were observed when the nsEP electrode was closest to the beam; the deflections diminished as the electrodes were moved further away. For the X+ plane (electrodes positioned to the right and parallel of the probe beam), the largest deflection signal was recorded between 0 and 100 μm away from the probe beam (Fig. 1A). For the Y+ plane (electrodes positioned above the probe beam), the largest deflection signal was recorded between 60 and 70 μm from the probe beam (Fig. 1B). Deflections of the probe beam tracked linearly with changes in the electric field (Fig. 1C) and in the pulse duration (Fig. 1D). The greatest deflections were observed at the highest electric field and with the longest pulse duration. These near-field deflections were undetectable below an electric field of 2.7 kV/cm or a pulse width of 30 ns. The time required for these deflections to return to baseline was long (>35 ms) suggesting that they could be thermal transients.

### Thermal Profile of nsEP

**Figure 2:**
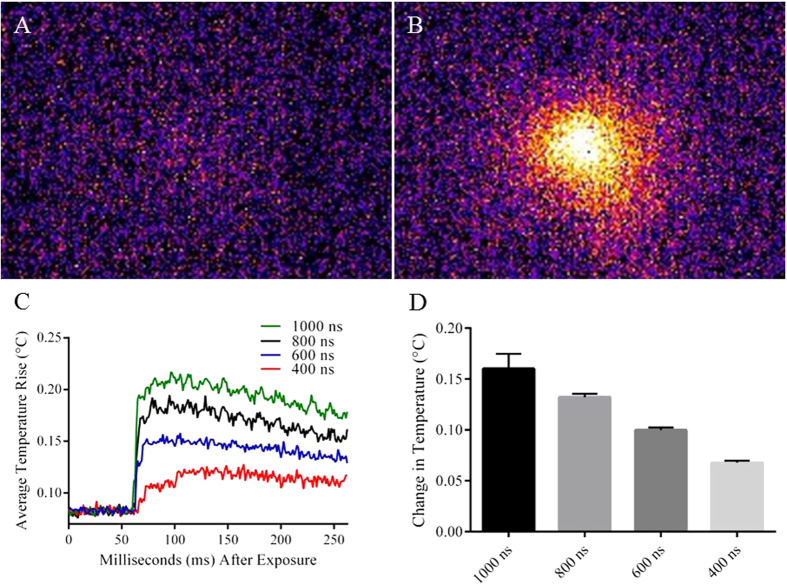
Thermal Profile of nsEP. (**A**) FLIR image of nsEP electrodes before the application of a single nsEP pulse. (**B**) FLIR image of nsEP electrodes 1.25 ms post exposure. (**C**) Traces of average temperature rise at the electrodes with either 1000, 800, 600, or 400 ns pulse delivered (**D**) Average change in temperature with either 1000, 800, 600, or 400 ns pulse. Error bars represent the standard error of the mean (SEM). A single 600 ns pulse causes a temperature increase of approximately 0.1 °C at 1.25 ms post exposure. Tukey’s multiple comparison test was performed, each data set was found to be significantly different form each other. Siginificance was not noted on the figure for simplificaiton purposes. P-values, 1000 ns vs. 800 ns(<0.005), 1000 ns vs. 600 ns (<0.000005), 1000 ns vs. 400 ns (<0.000005), 800 ns vs. 600 ns (<0.005), 800 ns vs. 400 ns (<0.000005), and 600 ns vs. 400 ns (<0.005). Note, the FLIR camera used for this experiment has a frame rate of 800 frames/second, therefore the initial maximal temperature spike may not have been captured.

Due to the nature of the waves detected by PBDT very near the electrodes, infrared thermography was performed in an effort to determine the total increase in thermal energy deposited by a typical nsEP pulse. Pulse durations of 1000, 800, 600, and 400 ns were used at 1000 V (applied), yielding an electric field of 13.1 kV/cm at the imaging plane. Figure 2A,B show a colorized FLIR image of the electrodes 1.25 ms before and after the nsEP pulse. An average thermal profile for each of these pulse durations is plotted in Fig. 2C. The 1000 ns duration pulse caused an increase of approximately 0.15 °C, whereas 800 ns, 600 ns and 400 ns pulse durations, caused an increase of 0.13, 0.1, and 0.075 °C respectively. The speed of the camera, at 800 frames/sec limited our ability to detect thermal increases occurring much sooner after or during the pulse.

### Detection of Far-field Waves Produced by nsEP Using PBDT

**Figure 3:**
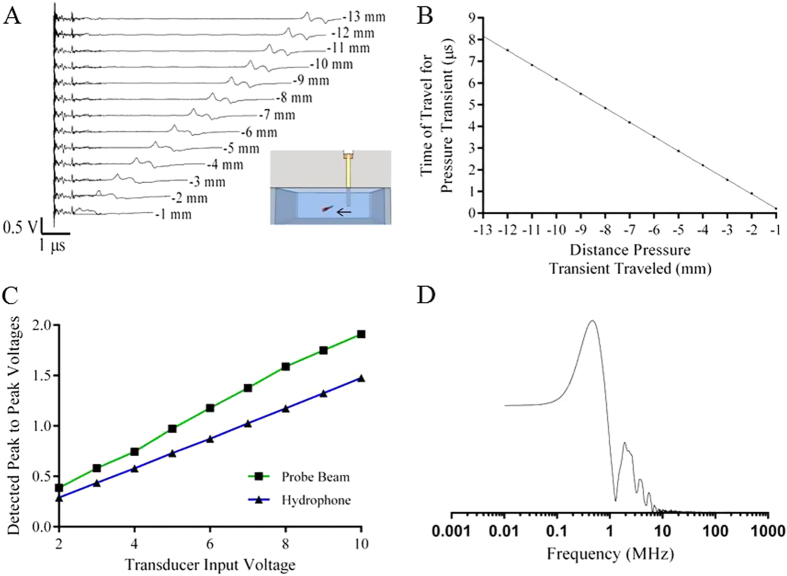
Characterization of the pressure transients generated by nsEP and detected by PBDT. (**A**) This figure shows the relationship between the distance of the nsEP electrode from the probe beam and the time required for the resultant pressure transients to interact with the probe beam. The nsEP electrodes were scanned from right to left for 12 mm in 1 mm steps. The inset figure shows the relative location and movement of the nsEP electrode. (**B**) The time at which the maximum peak of each pressure transient occurred was plotted against the distance the nsEP electrodes were from the probe beam. Using the slope of this graph, we calculated the speed of these pressure transients to be 1511 m/s. Linear regression analysis was performed (Y = −0.6615*X - 0.4432, R^2^ = 0.9952). (**C**) This is a calibration curve for both the PBDT and hydrophone. Using the calibrated Onda hydrophone and the provided data sheets we were able to calculate the voltage/pressure relationship for the PBDT to be 15 μV/Pa. (**D**) Fast Fourier transform (FFT) of the PBDT signal captured for a representative pressure transient. *Drawing in Figure A was drawn by CCR.*

In the far field (>1 mm away from the probe beam), we identified deflections in the microsecond time domain. The nsEP electrodes were scanned in 1 mm increments in the X+ (Fig. 3A), X- (Fig. S1) and Y+ planes (Fig. S2) and the PBDT signals were captured. The time-delay between the application of the nsEP pulse and the resulting deflection corresponded linearly with the distance between the nsEP electrodes and fixed probe beam. This time delay was due to the travel time of the induced wave, the speed of which was determined by plotting the travel time of the wave against the distance of the electrodes from the probe beam (Fig. 3B). The speed of the phenomenon was found to be 1.511 mm/μs, which is very close to the speed of sound (c_s_ = 1.5023 mm/μs) in normal saline at 23 °C^24^. Based on the speed at which these waves travel we identified them as acoustic pressure transients.

To determine the pressure generated by the nsEP, we used an ultrasonic transducer to generate a positive control pressure transient. The peak to peak voltage changes recorded by the PBDT and a co-localized calibrated hydrophone were plotted as a function of transducer input voltage (Fig. 3C). The generated pressure was then determined from the calibrated Onda hydrophone calibration and related to the probe beam deflection voltage, which was determined to have a sensitivity of 15 μV/Pa. We then indirectly quantified the amount of pressure produced by a typical nsEP exposure (600 ns, at 13.1 kV/cm). Supplementary Fig. 3 shows the calibration setup and the pressures generated in this experiment can be found in supplementary Table 1. We calculated the peak pressure at 5 mm from the electrodes to be 13 kPa for a 13.1 kV/cm, 600 ns pulse.

To ensure that the PBDT had sufficient frequency response to capture the waves produced by nsEP, we performed a FFT for the transducer signal captured by PBDT (Supplementary Fig. S4) and compared it to the FFT of the same signal captured by the calibrated hydrophone (Supplementary Fig. S5). The frequency response of the signals from each of these techniques matched quite well and had a cutoff frequency of approximately 20 MHz. To quantify the frequency characteristics of nsEP-induced pressure transients, a FFT was performed on a representative 600 ns, 13.1 kV/cm nsEP and on the resulting PBDT signal. The FFT of the nsEP trace showed a broad frequency range with a peak at 1 MHz (Supplementary Fig. S6). The fundamental ultrasound frequency of the nsEP pressure transient was found to be approximately 2.5 MHz (Fig. 3D). Therefore, the nsEP-induced pressure transients were well within the pass-band of the PBDT system implemented in these experiments.

### The Effect of Altering the Electrical Parameters of the nsEP on the Pressure Transients

**Figure 4:**
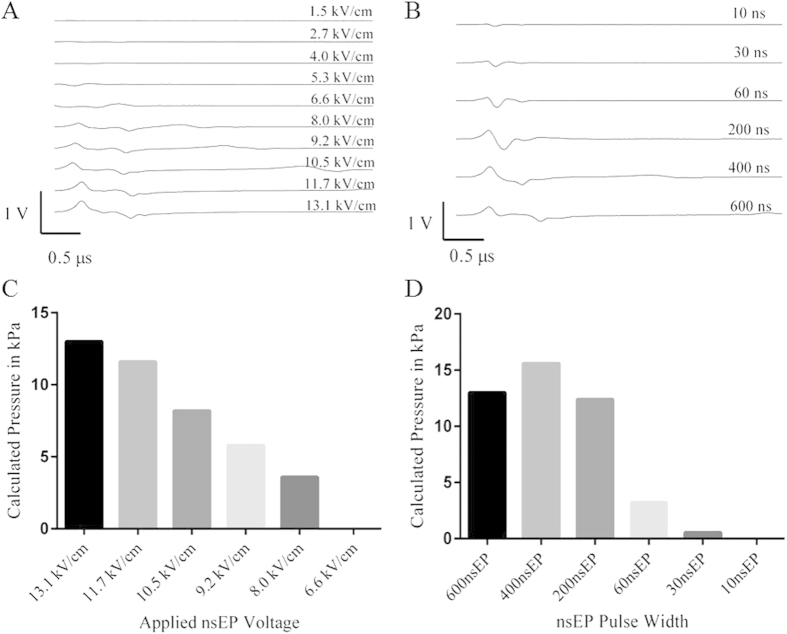
Influence of electrical pulse parameters on pressure transients. (**A**) At 600 ns and at a fixed distance of 5 mm in the X+ plane, the input amplitude of the nsEP was modulated in approximately 1.25 kV/cm steps. The greatest deflections were recorded at the highest electric field (13.1 kV/cm). It is interesting that the time between the positive peak and the negative peak of the measured pressure transient is approximately 600 ns in duration. (**B**) At a constant electric field of 13.1 kV/cm and at a fixed distance of 5 mm in the X+ plane, the pulse length of each nsEP was varied. The amplitude and shape of each deflection is different and is most likely due to the duration of the nsEP. (**C**) This graph displays the calculated peak pressures in kPa for the amplitude ramp in 4A. (**D**) This graph plots the calculated peak pressures in kPa for the pulse width ramp in 4B. Interestingly the 400 ns electrical pulse produced a pressure transient with 15.6 kPa of pressure, 2.6 kPa more than the longer pulse width of 600 ns.

Having determined that the deflections in the far-field from the electrodes were most likely pressure transients emanating from the nsEP electrodes, we decided to determine how the nsEP pressure transients depended on the electrical parameters used to produce the nsEP (i.e., the electric field and or pulse duration). At a fixed distance of 5 mm to the beam in the X+ plane, we recorded the deflections for 600 ns pulses at electric fields ranging from 13.1–1.5 kV/cm. Deflections recorded in the Y+ for same pulses can be found in supplementary Fig. 7. Altering the applied input voltage to the nsEP exposure changed the intensity of the electric field at the electrodes. At 1.5–4.0 kV/cm, no pressure transients were detected, suggesting a threshold for formation (Fig. 4A). At the higher electric fields, 5.3–13.1 kV/cm, deflection of the probe beam was observed with dependence in amplitude on the electric field, indicating that the pressure transients responded linearly with electrical input (Fig. 4A). These deflections occurred at approximately 3.3 μs after the pulse was fired, closely matching the time required for sound to travel 5 mm. The width of these initial deflections was approximately 600 ns. A secondary deflection can be seen trailing the first major set of deflections, possibly a reflection from an internal surface in the experimental tank. Rotating the electrodes 90° to the probe beam had no effect on the PBDT pattern or amplitude (supplementary Fig. 8A–D).

Using the same probe-beam and electrode orientation as in Fig. 4A, we altered the pulse durations from 10 to 600 ns, while holding the electric field at a constant 13.1 kV/cm. The smallest (lowest amplitude) pressure transient was detected at 10 ns and the largest (highest amplitude) occurred at 400 ns pulse width (Fig. 4B). These same measurements in the Y+ plane are shown in supplementary Fig. 9. Using the calibration constant previously determined, we were able to calculate the amount of pressure produced by each nsEP exposure. The calculated pressures from Fig. 4A are plotted in Fig. 4C and they show a linear dependence with respect to the electric field of an nsEP exposure. Figure 4D displays the pressures for the pulse width experiment. Curiously, the shorter pulse width of 400 ns generates a higher pressure transient than the longer 600 ns pulse. This result could be an artifact caused by reduced signal quality of the 600 ns pulse due to electromagnetic interference with the recording apparatus. Despite this result, the linear dependence of the pressure wave magnitude on the applied voltage to generate the nsEP provides further evidence that nsEP produce acoustic pressure transients.

### Schlieren Imaging of nsEP Generated Pressure Transient

**Figure 5:**
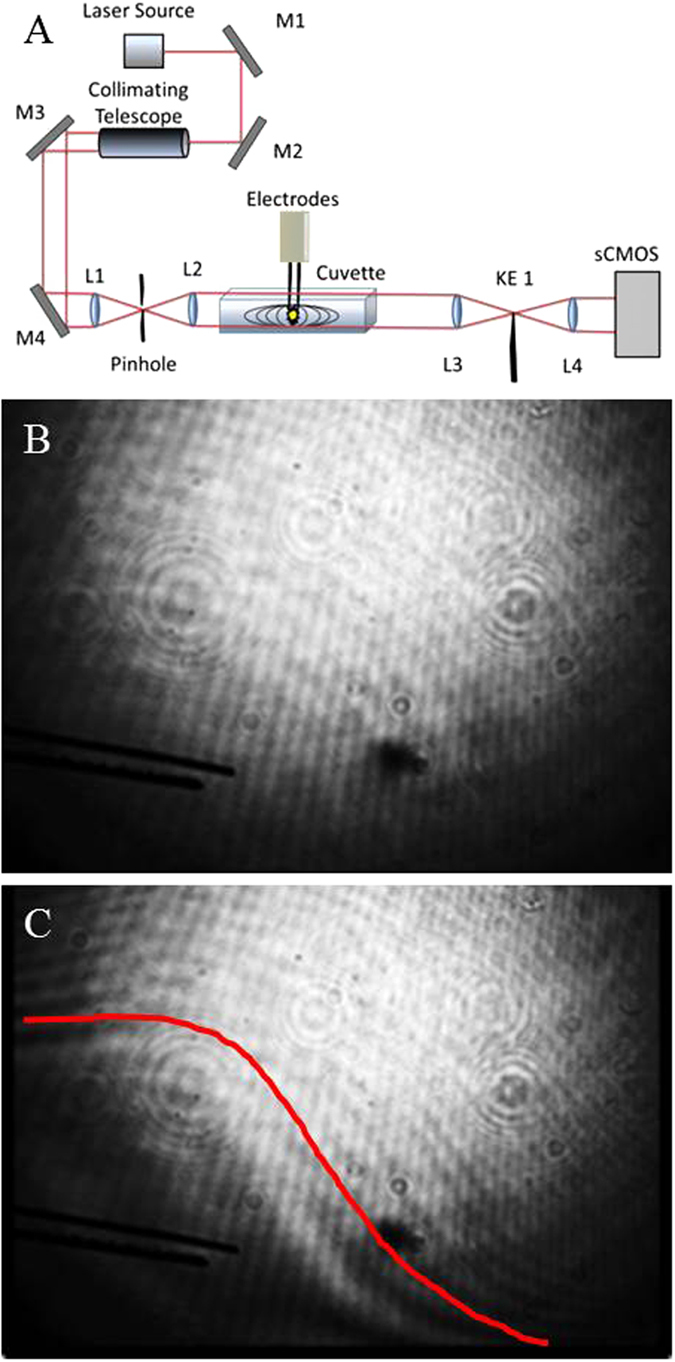
Schlieren image of the pressure transient. (**A**) Schematic of Schlieren setup with respect to the nsEP exposure setup. (**B**) Schlieren image taken before a single nsEP pulse, (**C**) Schlieren image of pressure transient immediately after a single 600 ns pulse at 13.1 kV/cm. The pressure transient (outlined by red line) can be seen, propagating away from the electrode. *Drawing in Figure A was drawn by LCM.*

To obtain further confirmation of a pressure transient produced by nsEP, the Schlieren imaging technique was used to capture an image of the pressure transient propagating away from the electrodes. The Schlieren imaging technique has the advantage of being able to capture changes in the refractive index of a media in two dimensions. In Schlieren imaging, collimated light passes through the area to be imaged before being focused onto an optical stop. Light that does not interact with a refractive index gradient will pass through the sample un-deflected and thus will be blocked by this optical stop. However, light that interacts with a physical wave in the image area will change direction and bypass the optical stop, where it is captured by a charge-coupled device camera to create a shadowgraph. A drawing of the Schlieren imaging setup is presented in Fig. 5A. Images before the pulse (Fig. 5B) and after (Fig. 5C) were taken and compared for the presence of a pressure transient. The pressure transient, outlined by a red line, can be seen immediately after the nsEP pulse in Fig. 5C, visually confirming the PBDT data.

### Pump-probe Laser Imaging of nsEP Electrode Pulsed at 600 ns

**Figure 6:**
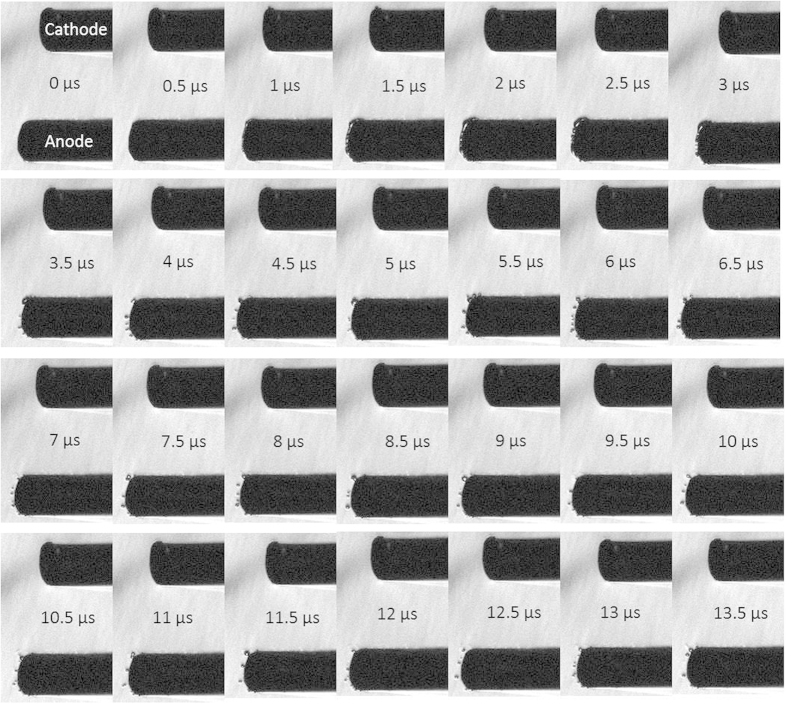
Pump-probe laser imaging of nsEP electrode pulsed at 600 ns and at 13.1 kV/cm. Collage of images captured at the time of the exposure (0 μs), during the pulse (0.5 μs) and after the pulse. A corona formed around the edge of the electrode (anode), at 1.5 μs after the initalion of the pulse. The corona existed for approximately 1.5 μs, eventually leading to the formation of microbubbles. These microbubble persisted for >10 μs.

Calculation of the speed of the pressure transients and comparison to the relative location of the nsEP electrodes, indicated that the pressure transient most likely emanated from the vicinity of the electrodes. The Schlieren image in Fig. 5C confirmed that the source of the pressure transients was the electrodes, thus we sought to visually capture any physical phenomena occurring at the electrodes during and after the pulse. Figure 6 is a collage of images collected beginning at the time of the exposure (0 μs), during the exposure (0.5 μs) and for several frames after. A corona can be seen forming around the edge of the electrode (anode), at 1.5 μs after the initiation of the pulse. This corona existed for approximately 1.5 μs, eventually leading to the formation of microbubbles. These microbubbles appear and cavitate >10 μs after the exposure. The number and the density of bubbles decrease with time, with fewer bubbles formed by 13.5 μs after the end of the nsEP.

### Effect of Pressure Transients on Nanoporation

**Figure 7:**
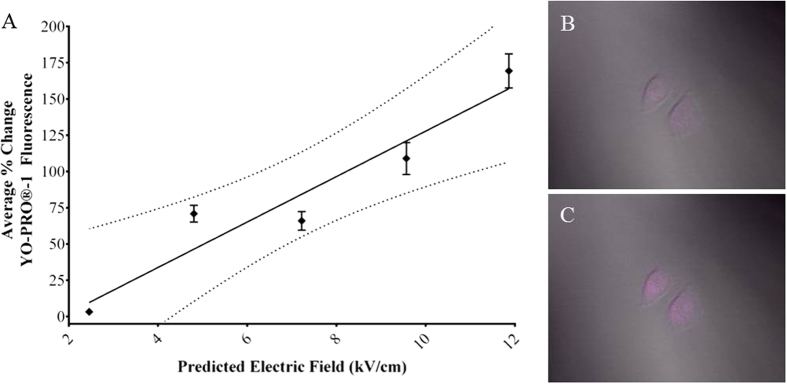
Effect of electric field on increase in YO-PRO®-1 fluorescence after nsEP exposure. (**A**) Nanoporation, as indicated by increases in YO-PRO®-1 fluorescence after nsEP exposure, increases linearly with increases in the electric field. The values are plotted as an average change in fluorescence intensity (error bar represent ± SEM). Solid line represents the best fit from a linear regression preformed in GraphPad Prism 6. The dotted lines represent the 95% confidence interval. (**B**) Image of CHO-K1 cells stained with YO-PRO®-1 before a typical nsEP exposure. (**C**) Image of same cells 25 seconds post exposure.

In the previous experiments, we showed that the electric field intensity directly influences the creation of pressure transients (Figs 1C and 4A). We used increases in YO-PRO®-1 fluorescence immediately after nsEP exposure as an indicator of nanoporation. YO-PRO®-1, a nucleic acid stain, has been shown to enter live cells exposed by nsEP, suggesting it enters the cell via nanopores^25^,^26^,^27^. YO-PRO®-1 fluorescence can be non-linear especially if the indicator enters the nucleus, however, in our experiments, we only recorded changes in YO-PRO®-1 fluorescence occurring <30 seconds after exposure, thus remaining in the linear range of the stain. We applied a single 600 ns pulse at 12.0, 9.6, 7.2, 4.8, or 2.5 kV/cm and recorded the relative change in fluorescent intensity of YO-PRO®-1 within exposed cells. We found that relative increases in YO-PRO®-1 fluorescence, correlated linearly with increases in the electric field (Fig. 7A). A representative CHO-K1 cell, exposed with electrodes positioned 50 μm above, can be seen in Fig. 7B just before the pulse, and 25 seconds after the pulse (Fig. 7C).

**Figure 8:**
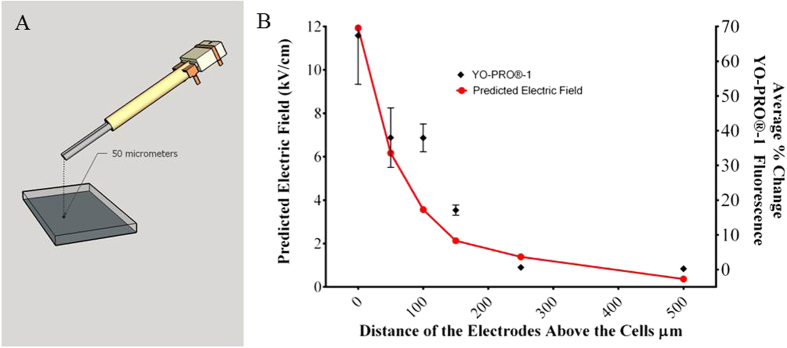
Effect of electrode height, relative to the cells, on YO-PRO®-1 fluorscence. (**A**) The majority of our typical nsEP exposures occur with the delivery electrodes being placed 50 μm above the cells to be exposed. This figure shows the relative position of the electrodes in a typical exposure. (**B**) We exposed CHO-K1 cells with a typical 600 ns pulse at 12 kV/cm and at varying electrode heights ranging from 0 μm to 500 μm above the cells. The predicted electric field, modeled by FEM is plotted in red on the left Y axis. Nanoporation, as indicated by the level of YO-PRO®-1 fluorescence change after the application of the nsEP (right Y axis) is plotted against the cooresponding height of the electrodes. The values are plotted as an average change in fluorescence intensity (error bar represent ± SEM). The level of nanoporation follows the intensity of the electric field. *Drawing in Figure A was drawn by CCR.*

To determine what effect the pressure transients in the far field have on nanoporation, we placed the electrodes at varying heights to assess the effect of the pressure transients in the near vs. the far field. Figure 8A shows the typical electrode orientation, positioned 50 μm above the cells. This orientation was used with heights of 0, 50, 100, 150, 200, 250 or 500 μm above CHO-K1 cells stained with YO-PRO®-1. A single 600 ns pulse at approximately 12.0 kV/cm was applied to cells at each height. The percentage of YO-PRO®-1 fluorescence increase was plotted vs time after application of the pulse (Fig. 8B). We determined the greatest level of nanoporation occurred when the electrodes were closest to the cells (near field). At 0 μm from the cells (the electrodes were touching the glass slide bottom of the dish) there was a 70% increase in YO-PRO®-1 fluorescence. YO-PRO®-1 fluorescence, and presumably nanoporation, dropped as the electrodes were moved away from the cells, indicating that the electric field maybe driving nanoporation either directly or indirectly. At 50 μm we observed an increase in YO-PRO®-1 fluorescence of approximately 40%. At 100 μm there was an increase of 36%, which dropped to 17% at 150 μm. Nanoporation, as indicated by YO-PRO®-1 did not occur with the electrodes at a height of 250 μm or higher above the cells. It appears that pressure transients, measured in the far field contribute little to the process of nanoporation.

**Figure 9:**
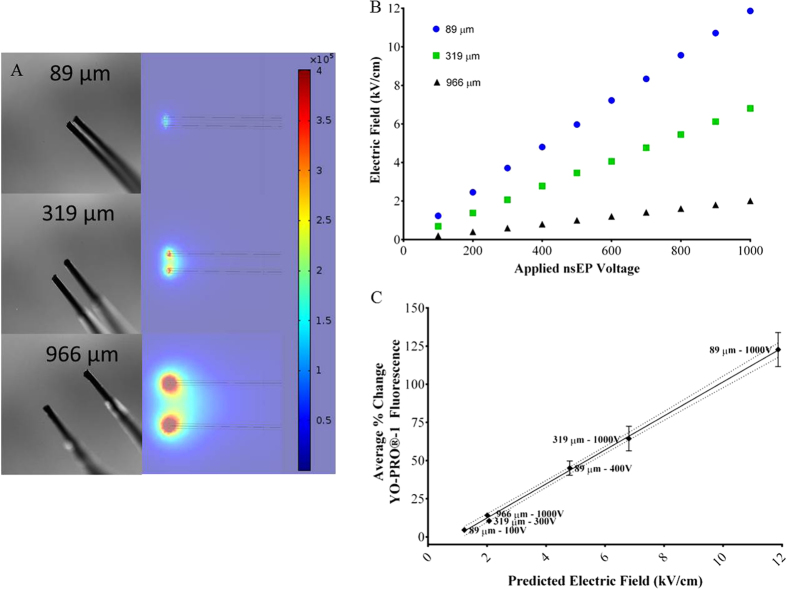
Effect of variations in electrode gap distance on nanoporation. (**A**) Bright-field image (4x objective, 40X total) of 3 pairs of electrodes with increasing gaps, 89, 319, and 966 μm. The predicted electric field as modeled by Comsol Multiphysics® appears on the right to each corresponding electrode. The color legend on the left shows the corresponding predicted electric field intensities. This model allowed us to adjust the applied voltage in order to achieve a similar electric field (approximately 2 kV/cm in the center) for each set of electrodes. (**B**) The Comsol Multiphysics® model predicted electric fields based on electrode gap distance and applied voltage. (**C**) Discrete voltages, as predicted by the Comsol Multiphysics® model in 9B were applied to each electrode pair to expose CHO-K1 cells, stained with YO-PRO®-1, with an equal electric field. Average increases in YO-PRO®-1 fluorescence were recorded and plotted (error bar represent ± SEM). Solid line represents the best fit from a linear regression preformed in GraphPad Prism 6. The dotted lines represent the 95% confidence interval.

In an effort to decouple electric field from the acoustic near field, we constructed electrodes with different gaps: 89, 319, and 966 μm (Fig. 9A). To account for differences in electrical field, we calculated the equivalent electrical fields for each electrode based on FEM model/simulation (Fig. 9A,B). 100, 300, and 1000 V were applied to the 89, 319, and 966 μm electrodes respectively, generating an electric field of approximately 2.0 kV/cm (100 V applied to 89 μm gapped electrodes yielded an electric field of 1.23 kV/cm, this was less than the desired 2.0 kV/cm. This is due in part to a drop in load with the smaller gapped electrodes). We performed a variety of exposures with the three electrodes and the changes in YO-PRO®-1 fluorescence closely tracked similar trends in the actual electric field. At an electric field of 1.23 kV/cm with the 89 μm gap electrode, we observed an approximately 5% increase in YO-PRO®-1 fluorescence. At electric fields of approximately 2.0 kV/cm, the 319 μm and 966 μm gapped electrodes produced a 10.5 and 14% increase in YO-PRO®-1 fluorescence respectively. Applying the maximum voltage of 1000 V to the 319 μm electrodes yielded an electric field of 6.8 kV/cm. Cells exposed using the 319 μm gap electrodes at 6.8 kV/cm yielded a 64.4% increase in YO-PRO®-1 fluorescence. Cells exposed by the 89 μm gap electrodes at 4.8 kV/cm yielded a 45.0% increase in YO-PRO®-1 fluorescence. The greatest increase in YO-PRO®-1 fluorescence occurred when 1000 V was applied to the 89 μm gapped electrodes, which consequently yielded the highest electrical field of 12.0 kV/cm. Changes in YO-PRO®-1 fluorescence mirrored the electric field trend, essentially, as the electric field intensity increased, so did the level of nanoporation.

## Discussion

Previous reports have shown that different cell lines have varying degrees of sensitivity to nsEP exposure. Adherent cells, like HeLa and CHO-K1 were found to be more resilient to the effects of nsEP than are suspension cell types such as Jurkat and U937^28^,^29^,^30^. These differences in viability were speculated to be related to the composition of each cell’s plasma membrane. To examine this hypothesis, Thompson *et al.* used atomic force microscopy to determine the Young’s Modulus for each of the cell types mentioned above^31^,^32^. It was determined that more rigid cell types had a higher threshold for damage by nsEP and thus had increased viability compared to less rigid cells. In a follow on experiment, Thompson *et al.* treated rigid cells with latrunculin A, (a sponge toxin capable of depolymerizing actin) thus making them “softer” and found that these cells became more prone to damage by nsEP^31^. These findings suggest that membrane rigidity could be a contributing factor for survivability of cells exposed to nsEP.

Further experiments have shown that altering the rigidity of the plasma membrane directly affects cellular viability when exposed to nsEP. Recently, we have shown that the depletion of cholesterol from CHO-K1 cells made them 50% more susceptible to nsEP exposure compared to sham-treated cells. Experiments with the trivalent cation gadolinium have shown that cells treated with this chemical agent and exposed to nsEP have a higher threshold for damage (higher viability) than do cells exposed in the absence of gadolinium^33^. Gadolinium, believed to make the plasma membrane more rigid, has been used as an MRI contrast agent and is used in electrophysiology to block sodium leak channels and stretch-activated ion channels (SAC). It is possible that the observed effect of Gd^3+^ is not entirely due to its ability to increase plasma membrane rigidly, but rather to its ability to block the mechanically sensitive SAC channels. Altogether, these studies show that alteration of the rigidity of a cell affects its sensitivity to nsEP. However, it is important to note that treating cells with toxic compounds, such as latrunculin and gadolinium potentially alters the cells normal response to nsEP, suggesting that generalized cellular stress may also contribute to the observed changes in susceptibility.

High speed calcium imaging has shown that nsEP causes a rapid increase in intracellular calcium that originates from membrane regions closest to the electrodes^34^. Beier *et al.* suggested the possible mechanism for the rapid increase in intracellular calcium is likely due to several mechanisms, including the formation of nanopores, the poration of intracellular organelles, and/or activation of specific ion channels^34^. It is possible that calcium enters the cell via mechanically activated channels or through the pore forming subunits of the piezo proteins found in cell membranes. Semenov *et al.* proposed that extracellular calcium via nanopores is a more efficient way of increasing intracellular calcium^35^. It is possible that a rapid increase in intracellular calcium could be caused by mechanical perturbation of the endoplasmic reticulum/plasma membrane stimulating the release of calcium from intracellular stores. This release of calcium could induce a cascade of channels to open, thereby allowing more calcium to flood into the cell. Interestingly, in a very recent publication, researchers using laser-induced cavitation as a high-throughput screening tool for mechanotransduction research, identified calcium release from the endoplasmic reticulum as a primary biomarker for cells exposed to a single intense shear stress wave^36^. These single intense shear stress waves, termed “μtsunami”, were also reported to directly or indirectly stimulate specific G-protein coupled receptors (GPCR) on the plasma membrane leading to the production of IP_3_^36^. Tolstykh *et al.* has shown that nsEP exposure activates the intracellular phosphoinositide signaling pathway^37^,^38^,^39^, hypothetically through the hydrolysis of phosphatidylinositol 4,5-bisphosphate (PtdIns(4,5)P_2_) or PIP_2_, a well-characterized intracellular pathway that originates on the inner surface of the plasma membrane. Hydrolysis of PIP_2_ ultimately causes intracellular calcium release from the endoplasmic reticulum via inositol trisphosphate (IP_3_) receptors, activating protein kinase C (PKC). The similarities between the observed bioeffects of a single intense shear stress wave (mechanical stimulation), and a single nsEP exposure are striking, and it is possible that the major biophysical mechanism behind nsEP action is due to mechanical stimulation.

In this paper, we present evidence of two different types of waves generated by nsEP exposure that could be responsible for the above mentioned mechanical stimulation. Waves emanating from the nsEP electrodes were recorded by PBDT as deflections both in the near-field and in the far-field. The waves differed in these two regions, offering clues as to the nature of biophysical mechanisms occurring after an nsEP exposure. The near-field deflections are thought to be thermal transients based on their limited spatial range, approximately four-fold larger amplitude compared to the far field deflections, and relatively slow rebound time (>35 ms). If this interpretation is correct, this result is an important finding because it provides evidence that the pressure transients generated by nsEP may be thermoelastic in nature, suggesting that rapid heating of the local environment by nsEP are responsible for the generation of pressure transients. Infrared thermography of the electrodes revealed a 0.1 °C temperature increase occurring 1.25 ms post 600 ns exposure. A 0.1 °C increase appears to be marginal; however, if this increase occurs within the span of a typical 600 ns, then this increase could be significant. A 0.1 °C rise over 600 ns would equate to a fast thermal gradient of 167,000 °C/second. Understanding the source of the pressure transients is fundamental to elucidating their potential biological effect; thus, these thermal waves warrant further study and characterization.

The deflections in the far-field are due to pressure transients interacting with the probe beam. The pressure transients traveled at the speed of sound, had a rapid relaxation time, and were not spatially constrained. Characterization of these pressure transients found that they have a peak frequency at 2.5 MHz and produce pressures in the 13 kPa range. Visualization of a pressure transient by Schlieren imaging revealed the Gaussian nature of the wave as it propagated outward from the electrodes. The finding that the pressure transient is Gaussian suggests the wave may be thermoelastic in nature and is the result of rapid heating of the solution around the electrodes during the exposure. With pump-probe laser imaging we observed the formation of a corona around the edge of the electrodes immediately after the nsEP exposure. Collapse of the corona resulted in many microbubbles forming randomly and persisting for 10 μs. This observation, the first of its kind for exposures used to induce nanoporation in cells, indicates that there is a mechanical component in the physical processes initiated by nsEP.

While pressure waves in the far field were clearly observed, they appear to have little impact on nanoporation when acting on the cells at distances greater than 250 μm. Our biological experiments imply that nanoporation tracks the intensity of the electric field. The stronger the electric field, the more nanoporation occurs. Data presented in Figs 7, 8, 9 corroborate these observations. When the electrodes are positioned 50 μm above the cells, increasing the electric field results in increases in nanoporation. Adjusting the height of the electrodes modulates the intensity of the electric field experienced by the cells. As the height of the electrodes is increased, the electric field intensity diminishes as well as the effect on nanoporation. These findings suggest that the electric field is either directly or indirectly responsible for nanoporation. We speculated that the microbubbles formed by nsEP exposure (captured in the collage presented in Fig. 6) could be responsible for nanoporation. It is known that the collapse of microbubbles can create jets, which, when near plasma membranes can cause damage, that appears similar to nanoporation. However, the microbubbles were only observed forming at or near the anodic electrode. To determine if the microbubbles played a role in nanoporation, we used electrodes with different gaps and examined cells in the middle of the electric field for nanoporation. Adjusting the input voltage to match the gap of the electrodes, ensured the production of electric fields of similar strength. No appreciable difference in nanoporation was observed with different gapped electrodes, suggesting once again that the electric field and not microbubbles is responsible for nanoporation.

Determining the effect of the pressure transients on nanoporation in the near field is much more difficult. The acoustic near field and the electric field are intimately linked, with the intensity of the electric field most likely determining the strength of the acoustic near field. Not only is the acoustic near field constrained by the electric field intensity, but it is also chaotic, with significant fluctuations in pressure intensity due to constructive and destructive interference of the multiple waves^40^. We speculate that both the near field and far field waves are evidence of an uncharacterized event occurring at the electrodes, driven by electric field intensity. This unidentified event could be responsible for some of the observed bioeffects associated with nsEP exposure. A possibility is that this event is the atomization of water, caused by the rapid alignment and breaking or bending of the water molecules by the intense but short duration electric fields created by nsEP. When an electric field intensity is high enough, the bonds holding water molecules together become stretched and or may break, resulting in the production of a shock wave. That shock wave would slow down as it propagated outward, eventually coalescing into an acoustic pressure wave, much like the pressure transients we have characterized in this paper. The rapid increase in temperature and pressure at the water/electrode interface, occurring as a result of the atomization, would lead to electrolysis of the water. The electrolysis of water would result in the production of hydrogen and oxygen gas, which would in turn cause the formation of microbubbles. These microbubbles would be very similar to the microbubbles we recorded in Fig. 6. The remaining free hydrogen and oxygen ions would recombine to form reactive oxygen species (ROS). Although we did not measure ROS production in this paper, ROS has been previously detected and described in response to nsEP exposure^41^. More work must be aimed at identifying the cause(s)/source (s) of the pressure transients identified by this paper. Identification of the event that leads to the production of these pressure transients will not only provide new details to how electrical pulses behave in an aqueous environment, but it may finally the answer the question of how electric fields cause the breakdown of plasma membranes^4^.

## Methods

### PBDT setup

**Figure 10:**
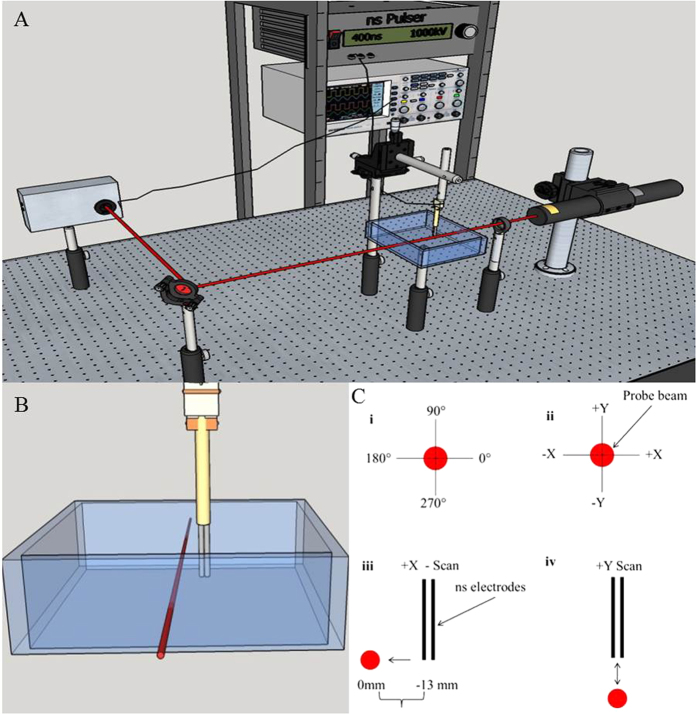
(**A**) Schematic of PBDT setup. The probe beam passes through our exposure tank containing approximately 350 ml of physiological buffer solution and is directed into our custom quadrature diode detector. The nsEP electrode is positioned parallel to and at a fixed distance from the probe beam. The electrodes can be moved in either the X-plane or in the Y-plane. (**B**) Close up view of nsEP electrode in relation to the probe beam. The nsEP electrode is mounted on two motorized stages and can move up to 25 mm in the X-plane and up to 12 mm in the Y-plane. (**C**) Shows the relative orientation of the nsEP electrodes to the probe beam, i) polar coordinates used, ii) probe beam coordinates, iii) scan in the X-plane, iv) scan in the Y-plane. *Drawings in Figures A–C were drawn by CCR.*

A 4.5 mW He-Ne laser (Thorlabs, Newton, NJ) with emission at 632.8 nm was employed as the probe beam. The laser was focused to a beam waist of approximately 150 μm for the fast axis of the beam, which was parallel to the nsEP electrodes as indicated in Fig. 10A. The probe beam passed through the center of a glass tank measuring 13.5 cm × 9 cm × 3 cm, containing approximately 350 ml of a physiological buffer comprised of 135 mM NaCl, 5 mM KCL, 10 mM HEPES, 10 mM Glucose, 2 mM CaCl_2_, and 2 mM MgCl_2_ (Fig. 10B). The buffer osmolality was 300 ± 10 mOsm and the pH was 7.4. After passing through the tank, the beam was reflected at a 45° angle into the custom-made quadrature diode detector. This quadrant silicon photodiode (Gamma Scientific, San Diego, CA) was chosen for its large active area (about 10 mm diameter) and fast response time. The nsEP electrodes were positioned in either +Y, +X, −Y, or −X planes (Fig. 10C) by a motorized stage capable of moving 25 mm in the X-plane and 12 mm in the Y-plane. When a wave generated from the electrodes intersected the probe beam, the variation in the refractive index of the medium caused the probe beam to deflect from its original direction, which appeared as an intensity and/or trajectory change in the output of the position detector^17^,^18^,^19^,^20^,^21^,^22^.

### Exposures/Data capture

The nsEP exposures were generated by a custom pulsing system previously described in the literature^42^. This custom nsEP pulser can deliver six discrete pulse widths, 600, 400, 200, 60, 30, and 10 ns with applied voltages ranging from 0 to 1000 V. The nsEP electrode was prepared similarly to previously published methods^17^, but, in short, the electrodes were constructed using 127 μm tungsten wire rods (A-M Systems, Sequim, WA). A single rod of the selected wire was then threaded through a piece of polyimide tubing (A-M Systems) only slightly larger than the wire (142 μm for 127 μm). Once threaded, two of the insulated rods were then threaded together through a borosilicate glass capillary (World Precision Instruments, Sarasota, FL) and fixed in place with superglue (Scotch Brand, 3M, St. Paul, MN). For each electrode, 12 mm of wire extended from the glass capillary, the last 6 mm of which were denuded of the polyimide coating. The gap between electrode rods was approximately 125 μm. Once the superglue was dry, the free ends of the electrode were connected to a type-K connector from OMEGA Engineering Inc. (Stamford, CT). Accurate delivery of the pulse was monitored on a Tektronix TDS-3054b e*Scope™ oscilloscope (Tektronix Inc., Beaverton, OR) for each pulse using a 100× high voltage probe. Each trace represented is an average of 180-200 traces collected over 3 min at a pulse rate of 1 Hz.

### Calibration of PBDT

The probe beam deflection technique was calibrated utilizing a calibrated Onda hydrophone (NC-1500, Sunnyvale, CA). A square wave pulse with varying input voltages was applied to an ultrasound transducer emission source. Both hydrophone and ultrasound transducer were submersed inside the same tank and buffer solution mentioned above. The transducer aperture was positioned such that the tangent vector to the aperture face pointed to the 45° surface of a right triangle prism positioned at the bottom of the tank. The aperture of the calibrated hydrophone faced the 45° slope of the prism from the left. The probe beam was focused to a point directly between the 45° prism surface and the hydrophone aperture. The focal point was positioned as close to the hydrophone aperture as physically possible, in this case less than 1 mm. The ultrasonic wave emitted from the transducer traveled down to the prism surface, reflected off of the prism and traveled parallel to the bottom of the tank towards the Onda Hydrophone aperture passing the probe beam along its trajectory. Signals were recorded from the hydrophone and the probe beam simultaneously on the Tektronix TDS-3054b e*Scope™ oscilloscope.

### Schlieren Imaging

The Schlieren imaging technique used a 4.5 mW He-Ne laser (Thorlabs, Newton, NJ) as the light source and a Zyla 5.5 sCMOS high speed camera (Andor, South Windsor, CT) with a 10 μs exposure time at 100 frames per second to record the resultant changes in the refractive index. Timing was accomplished by using a Stanford Research Systems digital delay generator (Sunnyvale, CA). This digital delay generator was used to trigger the nsEP and the subsequent imaging by the camera at different iterations after the pulse. The same glass tank mentioned above containing the same physiological buffer was used as the liquid medium for the propagation waves from the nsEP electrodes. The pulse duration was 600 ns at 1000 V using the previously described electrodes, yielding an electric field of 13.1 kV/cm. Captured data was analyzed with ImageJ^43^,^44^.

### Pump-probe Laser Imaging

We constructed a pump-probe laser imaging system to visualize the acoustic and thermal waves at the electrode surfaces, as well as effects on the electrodes themselves. The system consisted of a 70 ns, 532 nm Nd:YAG pulsed laser as a strobe source synchronized to the nsEP pulse firing to provide “snapshots” of the waves at discrete points during and after the pulse. Timing was critical in order for the propagation of the waves to be observed as they originate from the electrode and travel across the solution. The visual shape of the energy propagation as well as the presence and properties of cavitation effects was determined by acquiring images of both the thermal and acoustic waves.

### Determination of nanoporation

Chinese hamster ovarian (CHO-K1) cells from ATCC (Manassas, VA) were grown according to supplier’s recommendation in F-12K medium supplemented with 10% fetal bovine serum (FBS) and 1% penicillin/streptomycin at 37 °C, 5% CO_2_, and 95% humidity. Approximately 6.5 × 10^3^ were plated on 35 mm glass bottom dishes coated with Poly-d-lysine (MatTek Corporation, Ashland, MA) and allowed to incubate overnight at 37 °C, 5% CO_2_, and 95% humidity. Twenty-four hours later the cells were washed with DPBS and stained with YO-PRO®-1 (Life Technologies, Grand Island, NY) which was added to 3 mL of the physiological buffer described in the above sections. The buffer containing YO-PRO®-1 was added to the cells, and then incubated at 26 °C for 20 minutes. Cells were exposed on the Zeiss LSM 710 as described in our previous publications^45^. YoPro1™ fluorescence data was analyzed using Fiji (ImageJ).

### Modeling of Electric Field

A Finite Element Method (FEM) modeling of electric field, based on the solution to Maxwell’s equations, was performed using Comsol Multiphysics®. A 3D geometry was assembled with two cylindrical tungsten electrodes, in a cube domain, filled with saline solution, with a conductivity of 1.35 S/m. Voltages were applied to one electrode, while the other electrode had an applied voltage of 0 V (ground potential). Applied voltage was arbitrary, as it is only the relative difference in voltage between electrodes that affects the electric field strength. A heterogenous mesh was applied to decrease the granularity of the solution, in areas of interest. The boundaries of the cube domain are considered a soft boundary condition, resulting in a nulled field at the cube boundary. A stationary solver was utilized, and the solution electric field was plotted utilizing “slice” visualization. Electric field strengths were calculated for each experimentally applied voltage and electrode distance, to maintain an approximately equivalent electric field at the area of interest for each experimental configuration.

## Additional Information

**How to cite this article**: Roth, C. C. *et al.* Characterization of Pressure Transients Generated by Nanosecond Electrical Pulse (nsEP) Exposure. *Sci. Rep.* **5**, 15063; doi: 10.1038/srep15063 (2015).

## Supplementary Material

Supplementary Information
