# Supplementary material for: Characterization of Pressure Transients Generated by Nanosecond Electrical Pulse (nsEP) Exposure
Source: Sci Rep. 2015 Oct 9;5:15063. doi: 10.1038/srep15063 (PMC4598730; doi:10.1038/srep15063)
Supplement: Supplementary Information [file srep15063-s1.pdf]

Characterization of Pressure Transients Generated by Nanosecond Electrical Pulse (nsEP) Exposure

Caleb C. Roth[1], Ronald A. Barnes Jr.[2], Bennett L. Ibey[3], Hope T. Beier[4], L. Christopher Mimun[5], Saher M. Maswadi[2], Mehdi Shadaram[2], and Randolph D. Glickman[6]

[1]School of Medicine, Dept. of Radiological Sciences, University of Texas Health Science Center San Antonio, 7703 Floyd Curl Drive, San Antonio, Texas, USA 78229

[2]Dept. of Electrical Engineering, University of Texas San Antonio, 1 UTSA Circle, San Antonio, Texas, USA 78249

[3]Radio Frequency Bioeffects Branch, Bioeffects Division, Human Effectiveness Directorate, 711th Human Performance Wing, Air Force Research Laboratory, 4141 Petroleum Road, JBSA Fort Sam Houston, Texas, USA 78234

[4]Optical Radiation Bioeffects Branch, Bioeffects Division, Human Effectiveness Directorate, 711th Human Performance Wing, Air Force Research Laboratory, 4141 Petroleum Road, JBSA Fort Sam Houston, Texas, USA 78234

[5]Dept. of Physics, University of Texas San Antonio, 1 UTSA Circle, San Antonio, Texas, USA 78249

[6]School of Medicine, Dept. of Ophthalmology, University of Texas Health Science Center San Antonio, 7703 Floyd Curl Drive, San Antonio, Texas, USA 78229

Correspondence and requests for materials should be addressed to Caleb Roth (rothc@livemail.uthscsa.edu)

Supplementary Figures:

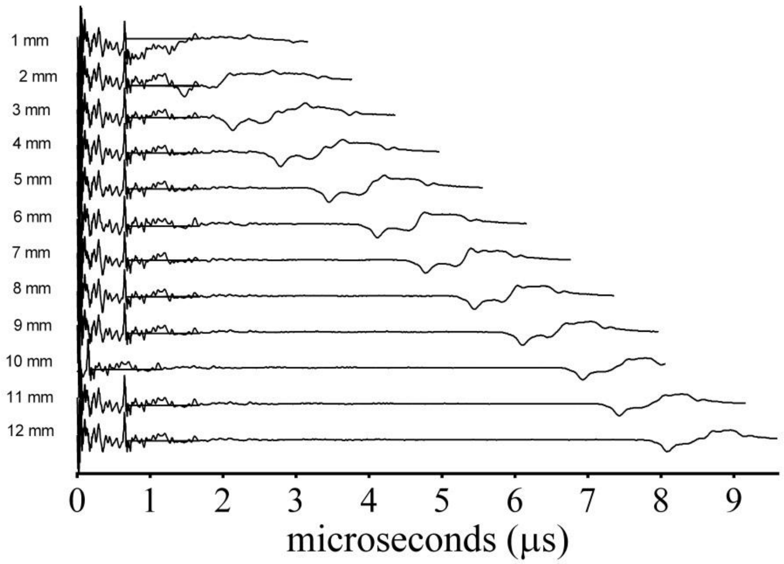

Supplementary Figure 1: Scanning the nsEP electrode in X- plane, and while maintaining the same anode/cathode configuration, the nsEP electrode was moved in 1 mm steps for 12 steps toward the beam. The resultant deflections were inverted compared to the traces recorded in Figure 3A.

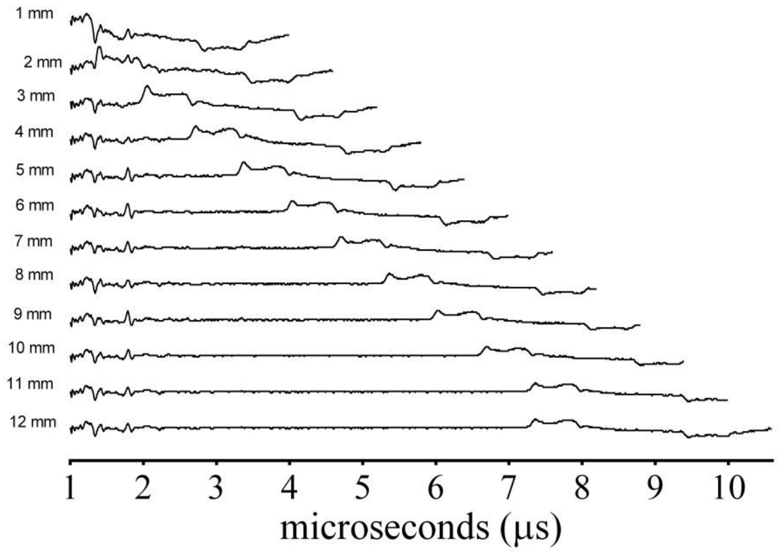

Supplementary Figure 2: Positioning the electrodes above the beam (Y+), the electrode was scanned in 1 mm steps for 12 steps away from the beam. The deflections tracked in time with the distance of the electrodes position above the beam.

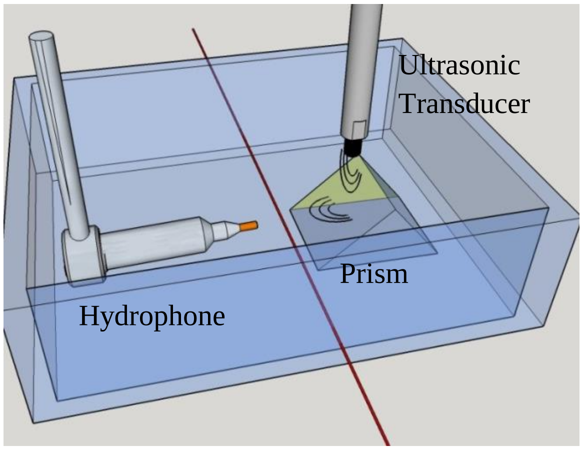

Supplementary Figure 3: Drawing showing the calibration setup and the relative locations of the ultrasonic transducer, prism, hydrophone, and probe beam. **Drawing was created by CCR.**

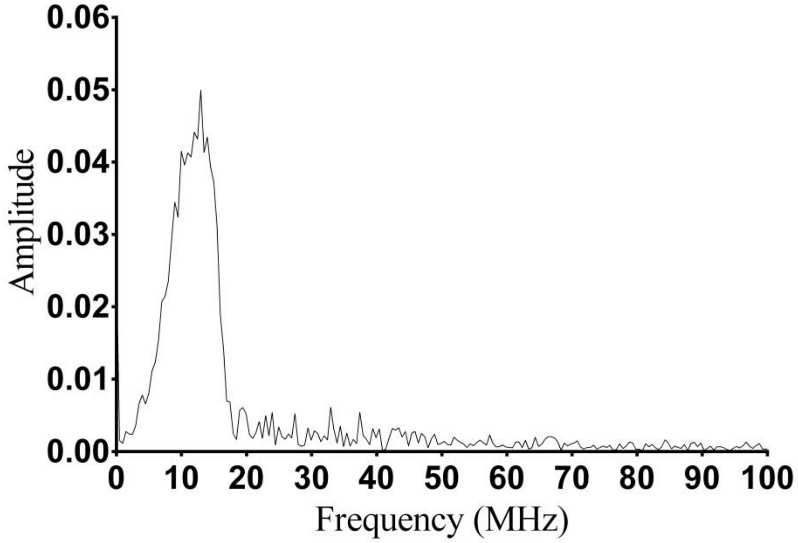

Supplementary Figure 4: FFT of transducer signal detected by PBDT.

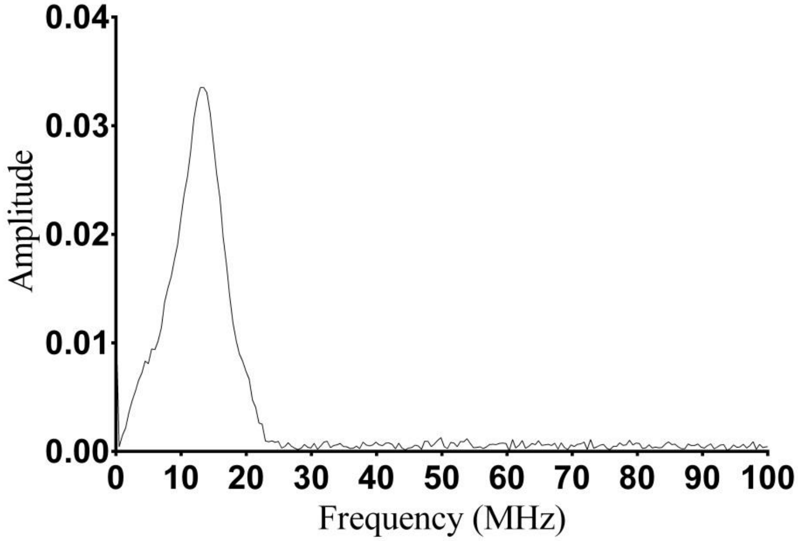

Supplementary Figure 5: FFT of transducer signal detected by hydrophone.

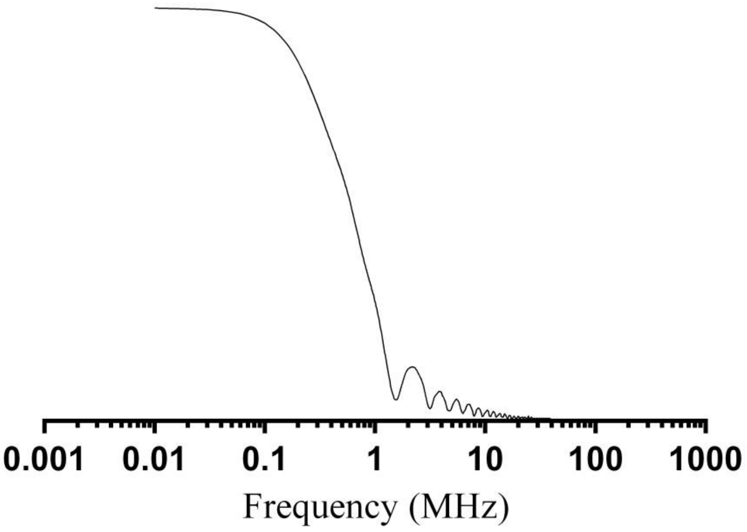

Supplementary Figure 6: Fast Fourier transform (FFT) of a representative nsEP pulse at 600 ns at 13.1 kV/cm and 20 ns rise time.

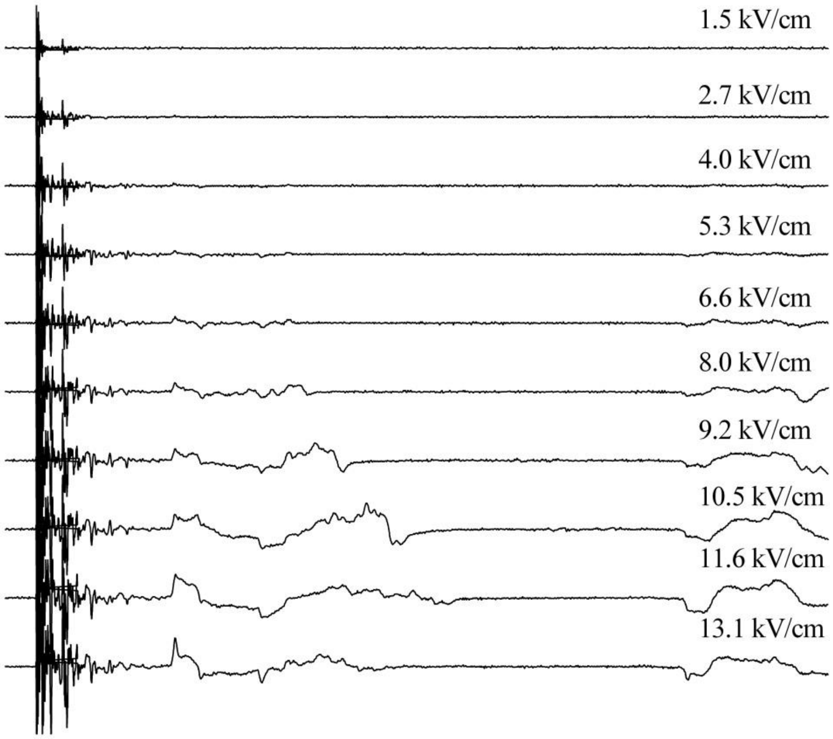

Supplementary Figure 7: At a fixed distance of 5 mm to the beam in the Y+ plane, we recorded the deflections for 600 ns pulses at electric fields ranging from 13.1 – 1.5 kV/cm.

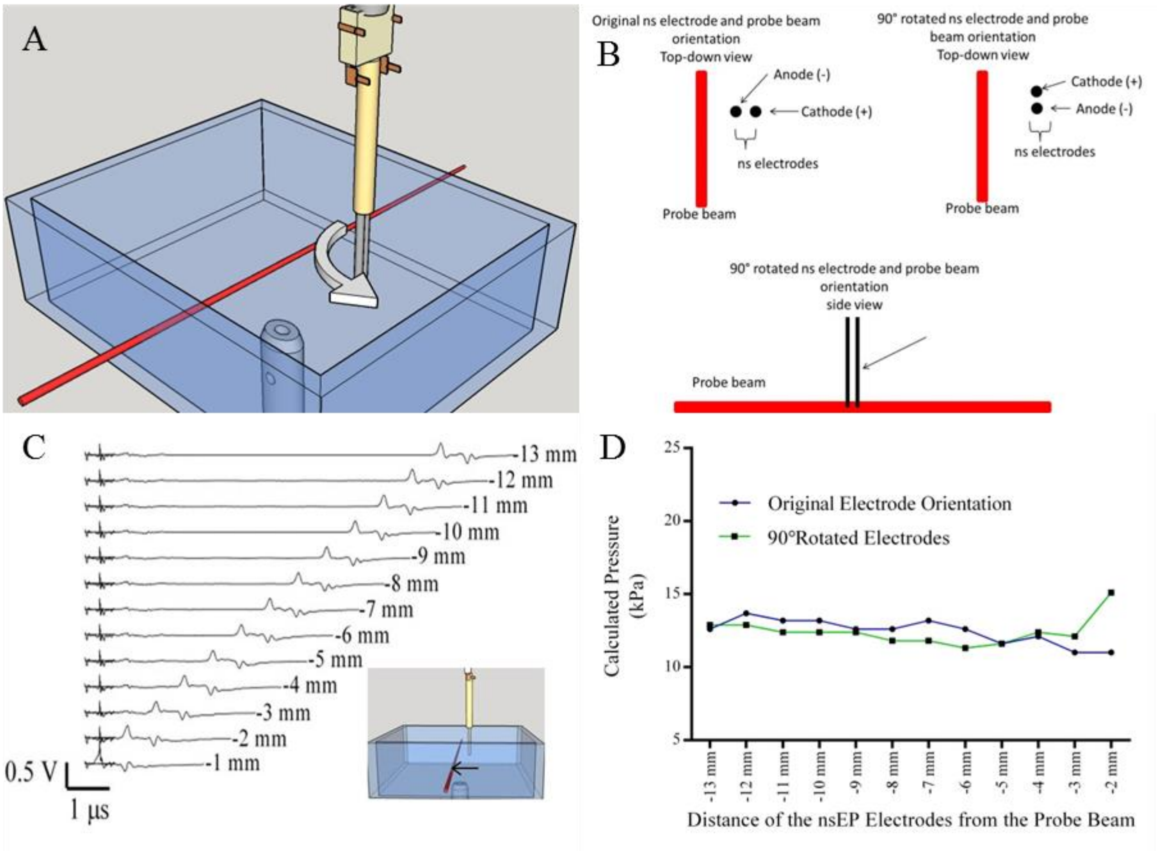

Supplementary Figure 8: Effect of rotating the nsEP electrode. A) Drawing showing the direction in which the nsEP electrodes were rotated. B) Diagram showing the rotation of the nsEP electrodes and the relative relationship to the probe beam. C) This graph shows the resultant deflections from the 90° rotated nsEP electrode as they were scanned from right to left for 12 mm in 1 mm steps. The inset figure shows the relative location and movement of the nsEP electrode. D) The calculated pressures for each pressure transient detected arising from nsEP electrodes placed at incremental distances from the probe beam. There was no difference between the calculated pressures, indicating the electrode orientation does not affect the production of the pressure transients. At -1 mm the nsEP electrodes interfered with the probe beam, making precise measurement of the pressure wave not possible. **Drawings A, B, and inset C were created by CCR.**

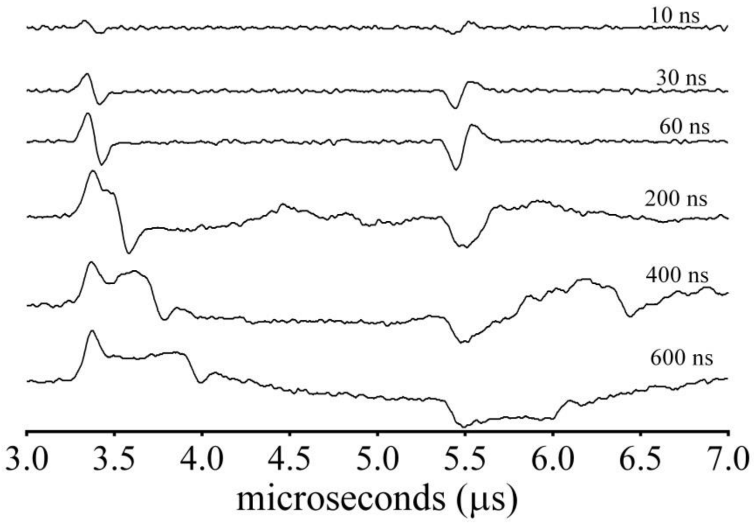

Supplementary Figure 9: At a fixed distance of 5 mm to the beam in the Y+ plane, we recorded the deflections for pulses ranging from 600-10 ns at an electric field of 13.1 kV/cm.

Supplementary Table 1:

Table 1: Calculated pressures from figure 3A.

Orientation of Electrode (Plane)	Distance of Electrode from Probe Beam (mm)	Pulse Width of nsEP pulse (ns)	Applied Voltage to nsEP pulse (V)	Pressure (KPa)
X+	13	600	1000	12.6
X+	12	600	1000	13.7
X+	11	600	1000	13.2
X+	10	600	1000	13.2
X+	9	600	1000	12.6
X+	8	600	1000	12.6
X+	7	600	1000	13.2
X+	6	600	1000	12.6
X+	5	600	1000	11.6
X+	4	600	1000	12.1
X+	3	600	1000	11.0
X+	2	600	1000	11.0
X+	1	600	1000	7.3
